# Gastrointestinal Cancer Patient Derived Organoids at the Frontier of Personalized Medicine and Drug Screening

**DOI:** 10.3390/cells13161312

**Published:** 2024-08-06

**Authors:** Zhenjie Yang, Jun Yu, Chi Chun Wong

**Affiliations:** 1 Institute of Digestive Disease and Department of Medicine and Therapeutics, State Key Laboratory of Digestive Disease, Li Ka Shing Institute of Health Sciences, The Chinese University of Hong Kong, Hong Kong, China; 1155113966@link.cuhk.edu.hk; 2 Institute of Digestive Disease and Department of Medicine and Therapeutics, Prince of Wales Hospital, The Chinese University of Hong Kong, Shatin, NT, Hong Kong, China

**Keywords:** gastrointestinal cancer, patient-derived organoids, drug screening, personalized medicine

## Abstract

Cancer is a leading cause of death worldwide. Around one-third of the total global cancer incidence and mortality are related to gastrointestinal (GI) cancers. Over the past few years, rapid developments have been made in patient-derived organoid (PDO) models for gastrointestinal cancers. By closely mimicking the molecular properties of their parent tumors in vitro, PDOs have emerged as powerful tools in personalized medicine and drug discovery. Here, we review the current literature on the application of PDOs of common gastrointestinal cancers in the optimization of drug treatment strategies in the clinic and their rising importance in pre-clinical drug development. We discuss the advantages and limitations of gastrointestinal cancer PDOs and outline the microfluidics-based strategies that improve the throughput of PDO models in order to extract the maximal benefits in the personalized medicine and drug discovery process.

## 1. Introduction

Cancer is the second leading cause of death globally. Gastrointestinal cancers account for about one-third of the global cancer incidence. Among gastrointestinal cancers, colorectal cancer (9.4%), liver cancer (8.3%), and gastric cancer (7.7%) ranked second, third, and fourth as the leading causes of global cancer-related deaths, respectively, in 2020 [1]. In the USA, the 5-year survival of patients with colorectal, gastric, liver, esophageal, and pancreatic cancer was only 65%, 32%, 20%, 20%, and 10%, respectively, for the period of 2010–2016. Besides, the efficacy of current therapies for patients with gastrointestinal cancer varies a lot [2,3,4,5,6,7]. This necessitates the development of personalized therapy and drug screening in patients with gastrointestinal cancer. However, cancer drug discovery in clinical trials often has high failure rates, mainly owing to the poor predictive power of existing preclinical models [8,9,10]. Although animal models have offered a huge contribution to the development of new medications, improved testing methods bridging the gap between preclinical animal models and the human body are urgently needed to better predict drug efficacy and safety in humans efficiently and reliably [11,12,13].

Recent studies have clearly demonstrated the utility of organoid models for drug screening [14,15,16]. Organoids are organotypic multicellular constructs generated from pluripotent or stem cells, which share similar structures and functions with their in vivo counterparts [17,18,19,20,21,22]. Patient-derived cancer organoids maintain the genetic features and heterogeneity of tumors of the parent tumor and remain genetically stable during long-term expansion, thus holding great promise for drug screening and personalized medicine [19,23,24,25,26]. Up until now, living biobanks of patient-derived organoids (PDOs) from a large number of tumors have been established [24,25,27,28,29,30,31,32].

In this review, we will cover the latest developments in gastrointestinal cancer PDOs and summarize the latest application of PDOs in personalized medicine and drug screening, their technical advantages, limitations, and future directions.

## 2. Establishment of Gastrointestinal Cancer PDOs

**Figure 1:**
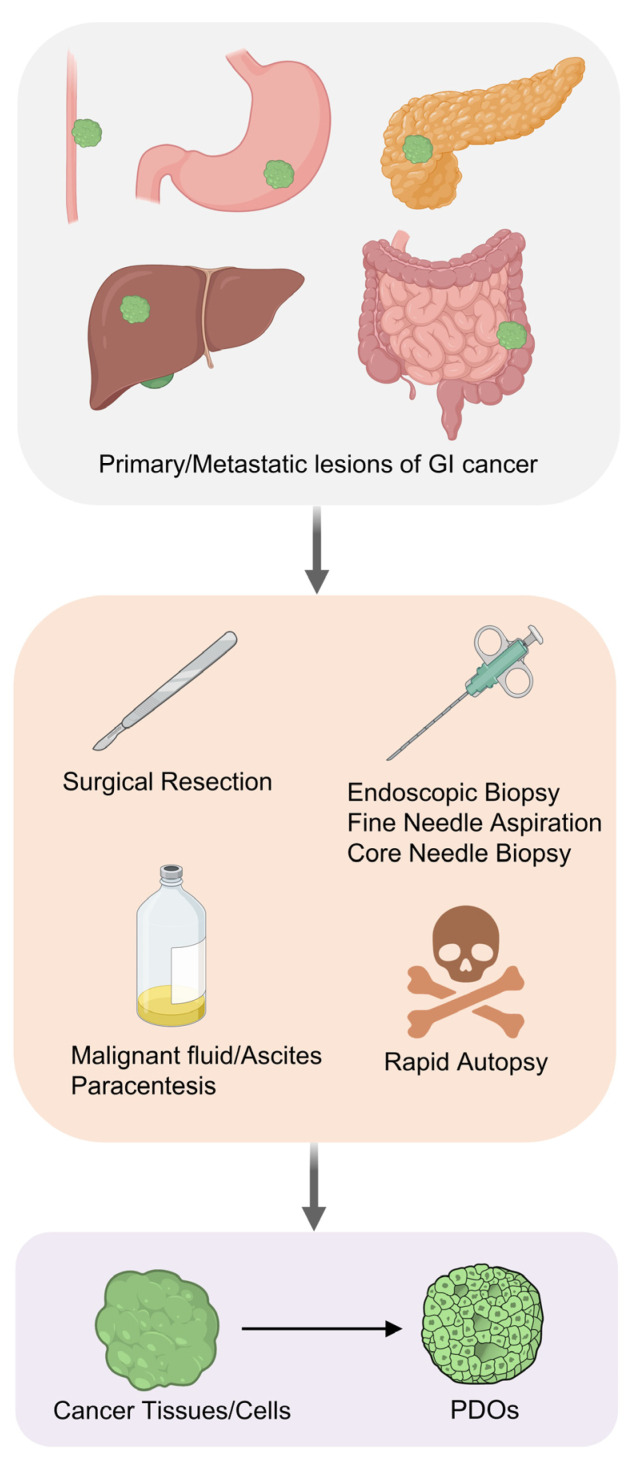
Gastrointestinal cancer PDOs could be generated from different kinds of specimens. Gastrointestinal cancer PDOs have been successfully established from specimens of surgical resection, endoscopic biopsy, needle biopsy or ascites paracentesis. Created with BioRender.com.

Protocols for the generation of gastrointestinal cancer PDOs are now well-established for different cancer types (Figure 1). The general establishment steps include: (1) removing non-epithelial tissue to purify the tumor tissues; (2) mincing the tumor tissues into small fragments with a scalpel; (3) enzymatic digestion to dissociate the tumor cells; and (4) cell plating and culturing in a 3D extracellular matrix hydrogel. Thus far, researchers have successfully established gastrointestinal cancer PDOs from samples obtained through surgical resection, endoscopic biopsy, ascites puncture, needle biopsy with an ultrasound or computed tomography guidance, and even rapid autopsy. As surgical resection is not a preferred treatment option for some patients with cancer, e.g., those with distant metastases, the realization to establish cancer organoids from endoscopic biopsy, needle biopsy, and ascites puncture overcomes a major limitation and facilitates the establishment of PDO biobanks in treatment-naïve patients during initial diagnosis or in patients receiving chemotherapy or radiotherapy without surgery. The maturation of organoid technologies, especially with the development of defined culture media formulations supplemented with specific growth factors and differentiation inhibitors, has greatly facilitated the maintenance of the stem cell niche, by promoting indefinite self-renewal and long-term proliferation [33]. In parallel, the analysis of the genomic landscape using high-throughput sequencing technologies (such as DNA and RNA sequencing) is frequently performed to elucidate the molecular characteristics of PDOs. Gastrointestinal cancer PDO libraries containing primary tumors, recurrent tumors, and distant metastases have been established worldwide, encompassing diverse cancer types with comprehensive molecular subtypes (Table 1 and Table 2). These PDOs could facilitate personalized therapy, drug screening, and the detection of chemoresistance in the clinic [19,24,34,35,36], as outlined below (Table 3).

## 3. Advances in Gastrointestinal Cancer Patient-Derived Organoids (PDOs)

### 3.1. Esophageal Cancer

Esophageal cancer consists of two major subtypes: esophageal adenocarcinoma (EADC) and esophageal squamous cell carcinoma (ESCC). PDOs from both EDAC [37,38] and ESCC [39] have already been successfully established. Li X, et al. successfully established EADC PDOs from tumor tissues retrieved during surgical resection with a success rate of 31%. Among these PDOs, 90% could propagate for over 6 months, except for those derived from well-differentiated tumors. The main causes of failure to establish culture include the lack of growth, bacterial infection, fibroblast overgrowth, or proliferative arrest [37]. In another report, Derouet MF, et al. also established 16 EADC PDOs from 28 endoscopic biopsy samples with a success rate of 57.2%. The primary issues limiting the successful establishment were bacterial contamination (21.4%) and the lack of growth (21.4%) [38]. Besides, Barrett’s esophagus cells have been identified as a potential source of contamination for EDAC PDOs. Barrett’s esophagus contains stem cells that grow well in a stem cell culture medium and harbor mutations similar to those of EDAC [38]. This necessitates the use of alternative approaches to eliminate the contamination of the Barrett’s esophagus epithelium in esophageal cancer PDOs. One approach involves using an organoid culture medium without gastrin, which allows the selective culture of esophageal cancer PDOs over Barrett’s esophagus cells [37]. Nevertheless, a clonality analysis based on copy number variation detection is a useful tool to validate the nature of the PDOs and to detect potential contamination from Barrett’s esophagus.

### 3.2. Gastric Cancer

The establishment of gastric cancer PDOs has been widely reported [40,41,42,43,60,61,62,63]. For example, Leung and colleagues have established gastric cancer PDOs encompassing all the major molecular subtypes as classified by The Cancer Genome Atlas (TCGA) (PMID: 25079317), including microsatellite instability (MSI), Epstein–Barr virus (EBV), chromosome instability (CIN, Intestinal), genomically stable (GS, Diffuse), and mixed subtypes. The majority (95%) of the established PDOs achieved a purity of over 90%, >85% of the frozen PDO stock maintained a good cell viability, and most of them could be passaged for more than 6 months without showing any decline in growth rate [41]. Nanki K, et al. demonstrated the successful culture of gastric cancer PDOs from surgical resection, endoscopic biopsy, and ascites puncture samples derived from either primary tumors or distant metastases. Even rare histological gastric cancer subtypes, such as signet ring cell carcinoma and hepatoid adenocarcinoma, can be propagated as PDOs in culture, thereby providing opportunities for the investigation of rare gastric cancers [42]. To selectively enrich gastric cancer PDOs from normal-like PDOs, Nanki K, et al. employed a selection strategy targeting dysregulated signals in human gastric cancer orderly, including (1) nutlin-3 as a marker to specifically enrich TP53-mutant tumors; (2) ROCK inhibitor free culture medium for RHO-dysregulated tumors; (3) TGFβ without A83-01 for TGFβ-insensitive tumors; and (4) EGF and FGF10 free culture media for tumors with constitutive growth receptor pathway activation. With the careful optimization of the positive selection protocols, the gross establishment efficiency has been enhanced from 54.7% (23/42) to 74.6% (44/59) [42].

### 3.3. Liver Cancer

Surgically resected tumor tissues from patients with primary liver cancer, including poor, moderate, or well-differentiated hepatocellular carcinoma (HCC), cholangiocarcinoma (CC), and patients with combined hepatocellular–cholangiocarcinoma (CHC), have all been utilized to establish organoids successfully [27,44]. Besides bulk tumors, Nuciforo S, et al. managed to generate PDOs from tumor samples obtained using a ultrasound-guided coaxial needle biopsy [28]. Their investigation proved that an ultrasound-guided coaxial needle biopsy technique was able to collect enough tumor samples to allow for adequate tissues for tumor organoid generation, clinical diagnosis, tumor staging, tumor marker identification, and whole exome/RNA sequencing [28]. Using this strategy, a PDO biobank was established, with PDOs derived from patients with liver cancer of various etiologies, including those associated with viral hepatitis, alcoholic liver disease, and nonalcoholic fatty liver disease [28]. Compared with other types of digestive cancer, the successful rate for the establishment of liver cancer PDOs is relatively lower (~20–30%). It has been reported that the success rate of liver cancer organoids was strongly correlated with the proliferation index of the original tumor [44]. PDO establishment success was 100% for those samples derived from tumors that contained >5% proliferating cells, whereas it was 0% for the samples derived from very-well-differentiated lesions with <5% proliferative cells [44]. The optimization of the culture protocol and medium formulation holds the key to further increase their success rate and unlock the full potential of liver cancer PDOs.

### 3.4. Pancreatic Cancer

Pancreatic cancer PDOs could be rapidly generated from resected tumors or biopsies and exhibit ductal- and disease-stage-specific characteristics [45,46,47,48]. PDOs have been established from pancreatic ductal adenocarcinoma (PDAC) and rarer subtypes, such as acinar cell carcinoma, squamous adenocarcinoma, and intraductal papillary mucinous neoplasm. Compared with the organoids of other digestive cancers, pancreatic cancer PDOs are relatively easy to generate with a higher success rate. Based on the distinct niche factor dependencies, three subtypes of pancreatic cancer PDOs have been identified: (1) Wnt non-producing subtype requiring Wnt for growth; (2) Wnt producing subtype autonomously secreted Wnt ligands; and (3) R-spondin independent subtype that grows in the absence of Wnt and R-spondin [48]. Besides, different organoid culture media compositions could be applied to functionally select pancreatic cancer PDOs with specific oncogenic mutations. Pancreatic cancer PDOs with wildtype SMAD4 were undetectable using IHC, because of the existence of the BMP/TGF-β-inhibiting molecules, Noggin and A83-01, in the organoid culture medium [47]. In contrast, the growth of SMAD4-mutant pancreatic cancer PDOs was unaffected by Noggin/A83-01 withdrawal. Thus, SMAD4 wildtype organoids can be distinguished from SMAD4 mutant organoids via functional selection by altering the culture media composition [47]. This finding highlighted the importance of selecting appropriate culture conditions for different applications.

### 3.5. Colorectal Cancer

Generally, colorectal cancer PDOs can be generated with a high efficiency from tumor resection specimens [24], as well as routine clinical biopsies [49,50]. Multiple CRC PDO living biobanks representing primary tumors and metastases have been established and characterized [49,50]. Van de Wetering M, et al. reported the establishment of PDOs derived from patients with colorectal carcinoma [24]. By refining the niche factor requirements, Fujii M, et al. established a PDO library encompassing diverse colorectal tumor grades and subtypes [18]. Apart from colorectal cancer, they generated organoids representing various subtypes of colon adenoma, a precursor lesion of colorectal cancer [18]. Liver is the most common site of metastasis in patients with colorectal cancer. Using ultrasound- or computed tomography (CT)-guided needle biopsy from liver metastases and pelvic metastases, a living biobank of PDOs from colorectal liver metastases has been successfully established [43]. This provided a powerful tool to study the pathology of colorectal liver metastases. For rectal cancer, Yao Y, et al. generated a living biobank with 80 PDOs derived from treatment-naïve patients with rectal cancer prior to surgical resection using endoscopic biopsy [51]. Although the samples of patients receiving systemic chemotherapy or radiation therapy are considered unlikely to yield viable cancer PDOs, Ganesh, K. et al. successfully generated 43 rectal cancer PDOs from patients who had undergone first- or second-line therapy chemotherapy and/or radiotherapy, as well as 22 rectal cancer organoids derived from treatment-naïve patients. Their rectal cancer PDO biobank included organoids generated from the distal, middle, and upper rectum, or sites of disease recurrence or metastasis [52].

### 3.6. Propagation of PDOs

Propagating methods are critical to PDO establishment, with tissue collection, digestion, and culture conditions being the most important variables. For the starting materials, samples from surgical resection generally have higher success rates compared with biopsy samples, as the quantity of tumor materials is higher. The next step in the preparation of PDOs is their dissociation into single cells, and the optimization of digestion time is an effective but often overlooked aspect of the protocol that can improve PDO propagation. For instance, the prolonged digestion time of liver surgery specimens (2–5 h) is desired to reduce the contamination of the normal tissue organoid [44]. For CRC, the treatment of digested tissues with trypsin releases more cancer cells that have infiltrated deeper into the intestinal wall [18]. Hence, the source of the materials and the processing steps have a significant impact on the success of PDO culture.

The optimization of the composition of culture medium is highly dependent on the tissue of origin of the cancers and their molecular subtypes. In CRC PDOs, Wnt activators (Wnt3A or R-spondin1), the p38 inhibitor (SB202190), and Noggin are often needed [18]. Whereas for liver cancer PDOs, R-spondin-1, Noggin, and Wnt3a are omitted from the medium formulation together, with the addition of dexamethasone and the Rho-kinase inhibitor to hinder non-tumoral outgrowth and contamination [44]. For GC, Nutlin3a can be added for enriching PDOs carrying p53 mutations, with a success rate of over 50% [41,64]. The influence of oxygen levels is also a subject of investigation. Hypoxia promotes the growth of a subset of CRC organoids [18]. By carefully adjusting the niche factors and oxygen levels, CRC organoids were propagated with 100% efficiency in one study [18]. However, a potential demerit is that the alterations of these factors may select for subclones that dominate the cultures.

### 3.7. Technological Advances in Gastrointestinal PDOs

Conventionally, PDOs consist of primary tumor cultures in 3D Matrigel, with the culture medium supplemented with various growth factors or pathway inhibitors depending on the tissue type. However, the typical Matrigel PDOs involve tissue dissociation and the encapsulation of single cells, which exclusively enrich cancer cells but fail to retain the tumor microenvironmental (TME) components, including stromal cells and immune cells [65]. One approach to overcome this issue is to independently harvest the other TME components, such as immune cells, that are subsequently co-cultured with organoids. To improve the PDO characteristics and better model TME, 3D air–liquid interface (ALI) culture, and microfabrication and microfluidic technologies have been utilized to establish PDOs.

3D ALI culture has been reported to support the in vitro cultures of minced gastrointestinal tissues [66]. In 3D ALI culture, minced primary tumor fragments containing both tumor cells and TME components are embedded in collagen gels within transwell inserts, with the culture medium added to the lower chamber. The top of the collagen gel is exposed to the air, allowing the cells sufficient access to an oxygen supply. ALI culture has been used to establish PDOs from a diversity of tumors, including colon, pancreas, lung, and kidney cancers. ALI culture could enhance the oxygen transfer to organoids and support TME cell populations. Both tumor parenchyma and stroma could be retained in ALI cancer PDOs, including fibroblasts and a variety of endogenous infiltrating immune cell populations [67]. ALI cancer PDOs preserving immune cells have been used to model the tumor-immune microenvironment and functionally recapitulate PD-1/PD-L1 immune checkpoint efficacy [67]. Whilst the immune component of ALI PDOs declines over time (could not develop beyond ~60 days), it provides a holistic strategy to explore the crosstalk between multiple distinct cellular populations. On the other hand, microfabrication and microfluidic technologies have greatly facilitated the high-throughput screening of gastrointestinal PDOs, which will be discussed in a later section. Continued advancements in PDO technologies will hold the key to more physiologically relevant PDOs that better predict drug responsiveness in human patients.

## 4. Gastrointestinal Cancer PDOs in Personalized Medicine and Drug Screening

### 4.1. Clinical Implication of PDO-Based Personalized Medicine and Drug Screening

Organoids are powerful tools in pre-clinical drug screening. Organoids derived from normal tissues or organ-on-chips that recapitulate more biomimical and complex biological parameters have been proven to be useful tools in single, double, or multiple organ toxicity and ADME (absorption, distribution, metabolism, and excretion) studies [68,69]. In particular, gastrointestinal cancer PDOs are highly suitable for personalized medicine and large-scale drug discovery, due to the ease of establishment of PDOs from tiny tumor fragments from biopsy, the preservation of original tumor phenotypes and genotypes, and the fact that they are relatively high throughput with short propagation times. Consequently, they reduce the waiting period for screening of targeted cancer drugs for cancer patients.

Gastrointestinal cancer PDOs bridge the gap between pre-clinical animal models and clinical trials during the drug discovery process. PDOs eliminate the species-specific differences and predict drug efficacy in humans more efficiently and reliably. PDOs also fill the gap between functional genomics and clinical drug response outcomes in the era of precision medicine. For instance, unlike other WNT pathway mutation organoids, such as adenomatous polyposis coli (APC)-mutant PDOs, the ring finger protein 43 (RNF43) mutant PDOs deliver a high sensitivity to Wnt secretion suppressors, since the RNF43 mutation cell is highly secreted WNT-dependency [24]. KRAS wildtype liver cancer PDOs are sensitive to the inhibition of EGFR-family blockers, whereas KRAS-mutant liver cancer PDOs showed intrinsic resistance [44]. Moreover, actionable key driver mutations are often lacking [70]. In a mixed cohort of patients with tumors, whole genome sequencing revealed that somatic alterations of 85.8% (660/769) sequenced samples were currently not targetable [71]. For these patients without specific actionable key driver mutations, large-scale drug screening in PDOs could also offer hope for patients. They have proven to be useful, time-saving, and cost-effective for the screening of therapeutic drugs. For example, ERK inhibition provided beneficial therapeutic effects in a subset of organoids that were insensitive to the BRAF and/or MEK inhibitors [44].

**Figure 2:**
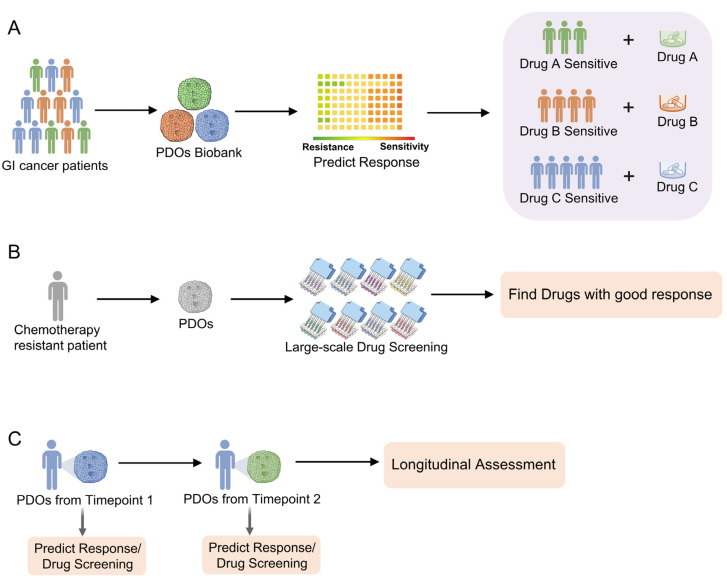
The application of gastrointestinal cancer PDOs for personalized medicine and drug screen. (**A**) With PDOs biobank, clinicians could predict drug response and resistance in advance and perform individualized cancer treatment. (**B**) For chemotherapy resistant patients, clinicians could perform large-scale drug screening and find drugs with good response. Existing drugs for treatment of other cancers or diseases might be efficient and repurposed. (**C**) PDOs enables longitudinal assessment of chemosensitivity in cancer patients. Clinicians could evaluate acquired resistance and chemosensitivity in time, and optimize personalized cancer treatment. Created with BioRender.com.

In summary, through co-clinical trials, the clinical trials conducted in both patients and the cancer organoids derived from them, clinicians could better predict drug response and resistance in advance and optimize individualized cancer treatment (Figure 2A).

### 4.2. PDOs for Precision Medicine and Personalized Treatment

Numerous studies have highlighted the clinical utility of gastrointestinal cancer PDOs for precision medicine and personalized cancer treatment [18,24,27,28,37,38,39,40,41,42,43,44,45,46,47,48,49,50,51,52,60,61,62,63,72]. Based on whole genome and transcriptome sequencing data from different studies, PDOs reflect the genetic features and heterogeneity of tumors of relevant patients and remain genetically stable during long-term expansion, thus holding great promise for personalized medicine [19,23,24,25,26,41]. For example, in a gastric cancer PDO biobank, the analysis of paired frozen cancer tissues and PDOs demonstrated that organoid cultures closely recapitulate in vivo tumors in terms of chromosomal stability/instability patterns. To investigate whether cancer PDOs with chromosomal instability could be maintained during long-term culture, they chose six long-term cultures of gastric cancer organoids and found that the chromosomal aberration pattern was stably maintained [41].

PDOs also maintain the spatially (intratumor and interpatient) heterogeneous responses to chemotherapeutics as in the primary tumors [27,37,46]. Therefore, through performing large-scale drug screening to identify drugs with a good response, existing drugs for the treatment of other cancers or diseases could be repurposed for chemotherapy-resistant patients (Figure 2B). For instance, liver cancer PDOs exhibited both obvious intratumor and interpatient drug response heterogeneity [27]. Anticancer drugs showed variable interpatient effects in a drug screening of 129 cancer agents tested on 27 liver cancer PDOs. A tyrosine kinase inhibitor, such as sorafenib, the first-line therapy for advanced liver cancer, showed divergent intratumor responses [27]. Among the six organoids derived from one patient “CCA8”, the survival rates after sorafenib treatment varied from 32.83% to 90.94%. This may partially explain the unsatisfactory effect of sorafenib in improving patient survival. Importantly, this study also identified effective drugs for individual PDOs for personalized oncology treatments. These results indicated that PDOs could guide and tailor treatment more accurately with optimal targeted anti-cancer therapies. We suggest that both intratumor and interpatient drug response heterogeneity should be considered when PDOs are utilized in personalized medicine. Therefore, for certain patients, multiple biopsies are necessary to ensure good representation of intratumor heterogeneity.

The ease of PDO establishment also enables the longitudinal and synchronous assessment of chemosensitivity in patients with cancer. A longitudinal follow-up with PDOs could reflect the clinical outcomes in an individual patient. With the help of PDOs, clinicians could evaluate acquired resistance and chemosensitivity in time, and optimize personalized cancer treatment (Figure 2C). For example, one patient with pancreatic cancer responded well to FOLFIRINOX (combination of oxaliplatin, irinotecan, fluorouracil, and leucovorin) and gemcitabine/nab-paclitaxel regimens. Consistently, initial PDOs derived from this patient at this time point, when patients are sensitive to chemotherapy, was sensitive to gemcitabine, paclitaxel, 5-fluorouracil (5-FU), and oxaliplatin. Approximately 2 years later, this patient presented with progressive disease, and PDOs cultured from this time point revealed the gene amplification of the KRAS allele and resistance to gemcitabine, paclitaxel, oxaliplatin, and SN-38 [46]. This case suggests the necessity of a longitudinal follow-up with PDOs to evaluate the acquired resistance of chemotherapeutics, which will facilitate precision medicine and personalized cancer treatment.

Thus, all these properties and advantages of PDOs provide the rationale for organoid-based precision medicine in the clinic. Doctors would be able to tailor patients’ treatment based upon drug-screening data from PDOs. The applicability of PDOs in optimizing cancer treatment has recently extended to the neoadjuvant setting. It has been reported that rectal cancer PDOs predicted the clinical response of neoadjuvant chemoradiation (NACR) in patients with rectal cancer [51]. The authors established 80 rectal PDOs from primary sites in treatment-naïve patients. After the patients received NACR, they compared the clinical outcomes with the responsiveness of the PDOs to chemoradiation in vitro, revealing that the PDO response correlates with patient outcomes with an AUC of 88% [51]. PDOs thus could help clinicians to determine which patients are sensitive to NACR.

Not only PDOs derived from primary sites, but also PDOs from metastatic sites could predict patient outcomes [43]. In a living biobank of PDOs derived from metastatic sites of pretreated patients with colorectal cancer and gastroesophageal cancer, Vlachogiannis G, et al. performed drug screening assays using a library of 55 anticancer drugs that are now in clinical trials or practice. A comparison of the efficacy of anticancer agents in PDOs with the responses of patients in clinical trials demonstrated 100% sensitivity, 93% specificity, 88% positive predictive values, and 100% negative predictive values, respectively, in predicting response to chemotherapy in patients [43].

### 4.3. PDOs for Large-Scale Drug Screening

Patient-derived organoids have the following advantages for drug discovery and testing. Compared with the cancer cell lines model, PDOs recapitulate the phenotypes and genotypes of the tumors better. PDOs could be established from different kinds of specimens, including surgical resection, endoscopic biopsy, needle biopsy, or ascites puncture. Thus, patient-derived cancer PDOs are increasingly applied in drug testing.

Multiple studies have demonstrated the utilization of PDOs for drug discovery [25,27,41,50]. In a gastric cancer PDO biobank, large-scale drug screening revealed a good response to unexpected drugs that were recently approved or in clinical trials for other cancer types, such as Napabucasin and Abemaciclib [41]. Similarly, drug screening in a liver cancer PDO biobank identified that Bortezomib, a proteasome inhibitor approved by the FDA for other cancers, was pan-effective across all liver cancer PDOs [27]. This highlighted the usefulness of PDOs in drug screening. Apart from monotherapy, the PDOs biobank could also be applied to evaluate drug combinations. In a colorectal cancer PDO library, the dual inhibition of the EGFR and MEK pathways showed a synergistic effect in suppressing the proliferation of RAS-mutant PDOs [25].

**Figure 3:**
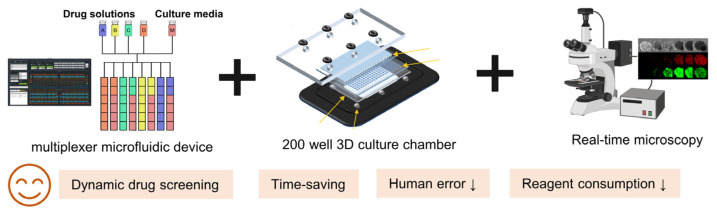
Main compositions of the automated microfluidic 3D organoid culture platform for dynamical drug screening of Schuster B, et al. [75]. With a programmed automated multiplexer microfluidic control device, this platform provides up to 20 different dynamic drug conditions, including monotherapy or combination therapy. Besides, this platform is high throughput with 200 chambers for organoid culture at one time. Their platform also enables synchronous analysis of organoids growth or apoptosis with real-time microscopy. The organoids in chambers could be harvested for additional analysis when an experiment is completed.

Although PDOs represent major savings in cost and time compared with patient-derived xenografts, they is still limited by their throughput in large-scale screening. For example, a 37 anticancer drugs screening in just one PDO with seven drug concentrations in triplicate would necessitates hundreds of data points, not to mention biological replicates and different organoid passages. This alone would require substantial investments in labor and time for conventional PDOs. However, the convergence of organoid culture, microfluidic, and sophisticated cell sensor technologies is promising to address these limitations in the drug discovery processes [34,73,74]. Schuster B, et al. have developed a robust and streamlined automated microfluidic platform with 200 individual chambers (Figure 3), allowing a high-throughput PDO culture, stimulation, assaying, and harvesting of organoids under dynamic conditions recently [75]. Most importantly, their platform facilitates dynamic programmed control of 20 independent fluidic conditions with a well-designed multiplexer control device, which is automatically controlled by solenoid valves and custom software, enabling the real-time screening of different sequences of drugs either individually or in combination. Once an experiment is completed, the organoids could be harvested for additional analysis [75].

The benefits of the automated microfluidic organoid culture platform in the assessment of drug safety and efficacy are as follows. Firstly, the use of microfluidics provides dynamical screens with individual drugs or cocktails, where the concentration, timing, and duration of the fluidic delivery can be precisely controlled in an automated fashion [74,76,77,78,79]. Besides, when integrated with an automated multi-sensor system [11], it could dramatically reduce the labor-intensive culture process, decease human error, and minimizes the reagents consumption, while being able to continuously monitor the cultures [80]. Furthermore, the precision and repeatability of mechanical automation offer the opportunity to reduce variability, potentially during the tedious experiment process [74,81]. Collectively, these developments highlight the promise of microfluidic platforms for high-throughput drug screening in PDOs.

## 5. Limitations of PDOs in Personalized Medicine and Drug Screening

Whilst PDOs present significant advantages over conventional cell line models in drug screening, several limitations remain. First, though most of the studies reported an excellent correlation between PDOs and clinical treatment response, two prospective clinical trials in small patient cohorts (SENSOR and APOLLO) produced mixed results in guiding treatment selection [82,83]. In the SENSOR trial, six patients received PDO-directed treatments, but none achieved an objective response. In the APOLLO trial, two patients received PDO-directed treatments, and one had a partial response. Second, the inability to guarantee the development of successful PDOs for all patients is another barrier in the clinical implementation of PDO-based drug screening, especially for liver cancer PDOs with a ~20–30% success rate. Third, although PDOs develop in a highly variable fashion through stochastic self-organization [74], there is still a lack of cytoarchitectural structure or microenvironment components (such as immune cells, stromal components, or blood vessels) that limit their physiological relevance [84]. Recent research has shown that PDOs co-cultured with other cellular components had a differential drug response [85,86,87]. Fourth, the predictive power of PDOs might be drug dependent. Ooft SN, et al. demonstrated that PDOs predicted the clinical response to irinotecan or the combination of irinotecan plus 5-FU/capecitabine; however, their PDOs failed to predict the outcome for a regimen of 5-FU plus oxaliplatin [50]. Fifth, patients with a limited lifespan might not be able to benefit from PDOs due to a longer turnaround time from biopsy to drug testing. Moreover, standard-of-care treatments after biopsy may alter tumor characteristics and therefore reduce the predictive accuracy of the PDOs [88]. To ensure that there is no major shift in drug sensitivity, it is important to establish PDOs from biopsies before or after therapy. Sixth, the standardization of PDOs is problematic. For example, the use of the Matrigel basement membrane extracts introduces unknown factors into the culture medium. This can be overcome with well-defined engineered hydrogels that are currently under development [89]. The use of different predetermined cutoffs to define drug response or resistance also complicates the data analysis and lab-to-lab variability. Finally, the throughput of PDOs is limited compared with the cell lines. To address these issues, combining the PDO culture with automated microfluidic and bio-sensor technologies may serve as a versatile and powerful pre-clinical tool for the drug discovery processes in vitro [34,73,74]

Future work in this field should optimize culture conditions, standardize assays, and raise throughput to achieve PDO-based precision medicine. By obtaining multiple core biopsies, and refining the culture medium and digestion protocols, the culture success rate can be further improved. The starting tumor material (number, size, and tumor/epithelial cell content of biopsy samples or resection specimens) is a critical factor for successful PDO establishment. The preselection of biopsies that contain sufficient tumor cells by a pathologist is an important method to improve the quantity and quality of the tumor material [43,83]. Besides, the anatomic location of the tumor tissue sample also needs to be considered, as intra-tumor heterogeneity may bring about changes in physiology and drug sensitivity.

## 6. Clinical Application of Organoid Technologies: From Bench to Bedside

A major challenge for the translation of PDOs is to ensure consistent quality in the clinical setting. For the clinical application of gastrointestinal cancer PDOs, it has to comply with the requirement of the Federal Drug Administration’s Current Good Manufacturing Practices (c-GMP) in order to guarantee the manufacture of high-quality products for patients. This includes the construction of specialized facilities for product development, product manufacture, quality control testing, and compliance with major regulatory bodies. All of these are critical for the clinical application of PDOs for personalized medicine and the drug screening process. For example, the establishment of personalized gastrointestinal cancer PDOs requires the rapid isolation of fresh cells. GMP facilities allow fresh tumor material to arrive at the manufacturing facility as soon as possible (within a few hours) from the operating room as this is essential. Hospital-based GMP facilities are recommended to ensure the rapid and optimal organization and transport of cellular material [90]. Besides, the adaptation of c-GMPs will help to reduce batch-to-batch variability [91], and improve the consistency, reproducibility [92], and viability of PDOs [93]. Doctors should also communicate with patients with regards to the expected benefits of organoid technologies so that informed choices can be made [94]. To overcome the hurdles, close and effective collaboration is needed between experts from various disciplines, including basic and clinical research, product development, manufacturing, quality assurance, quality control, and regulatory affairs [94].

## 7. Outlooks and Perspectives

The application of gastrointestinal cancer PDOs is rapidly evolving. Although numerous challenges remain to be addressed, these approaches are sure to have a positive impact on basic research and clinical translation. They are not only used for conventional and targeted chemotherapeutics screening, but they are also viable platforms for evaluating immune therapy efficacy, such as PD-1 blockade therapy [67,95] and combination therapy (such as the combination of immune checkpoint inhibitors and angiogenesis inhibitors) [96,97]. Besides, genetic editing in PDOs has allowed the selection, screening, and expansion of clones with the desired mutations in basic research and might be applied in the clinic in the future [98,99].

Another application of gastrointestinal cancer PDOs is to test the effect of probiotics or evaluate the effect of the microbiome on chemotherapy, which may serve as a discovery tool for the development of microbiome-related cancer therapeutics. It has been reported that gut microbes have been implicated in influencing responses to chemotherapy and immunotherapy in pre-clinical models and patient cohorts [100,101]. Initial studies have demonstrated the feasibility of PDOs in co-culture with microbial and viral components [75,102]. Currently, the co-culture of gut bacteria with PDOs is typically achieved by the direct microinjection of bacteria into the lumen of organoids [103,104]. PDOs harbor low intraluminal oxygen levels suitable for anaerobic bacteria growth, permit the assessment of bacterial infiltration and translocation in a 3D structure with nutrient and oxygen gradients, and better mimic gut microbes–epithelial cell interaction with heterogenous cell populations with varying polarity and differentiation [103]. While PDOs cannot entirely replicate the complex tumor microenvironment [105], it is a physiologically relevant model for the understanding of host–microbiome interactions, especially for gastrointestinal cancer PDOs.

With improvements in their success rate and increased throughput, PDOs are expected to benefit personalized medicine and drug discovery process in the near future.

## Figures and Tables

**Table 1:** Summary of patient-derived organoids in gastrointestinal cancers.

Cancer Type	Sample	Cell Type	Location	Success Rate	Maintenance	Ref.
Esophageal cancer	SR	EADC	PT	31% (10/32)	≥25 passages	[37]
Esophageal cancer	EB	EADC	PT	57.2% (16/28)	N/A	[38]
Esophageal cancer	EB	ESCC	PT	71.4% (15/21)	N/A	[39]
Gastric cancer	SR	Adenocarcinoma	PT ^f^	N/A	≥1 year	[40]
Gastric cancer	SR	MSI; EBV; CIN; GS	PT; M	over 50%	≥6 months	[41]
Gastric cancer	SR; EB; Paracentesis	MSI; CIN; GS	PT; M; Ascites	74.6% (44/59)	≥3 months	[42]
Gastric cancer	EB; US-guided NB; CT-guided NB	N/A	M	N/A	N/A	[43]
Liver cancer	SR	HCC; CC; CHC	PT	44% (8/18)	≈1year	[44]
Liver cancer	SR	HCC; CC	PT	N/A	N/A	[27]
Liver cancer	US-guided NB	HCC; CC; LEL-CC	PT	26% (10/38)	≥32 weeks	[28]
Pancreatic cancer	SR; FNA	PDAC	PT; M	80% ^a^; 100% ^b^	≈20 passages	[45]
Pancreatic cancer	SR; FNA; Rapid autopsy	PDAC	PT; M ^g^; Ascites	78.2% ^c^; 71.6% ^d^; 45% ^e^	≥5 passages	[46]
Pancreatic cancer	SR; FNA	ACC; PDAC; Adenosquamous PDAC; IPMN-derived PDAC	PT	62.7% (52/83)	N/A	[47]
Pancreatic cancer	SR; FNA; Paracentesis	PDAC	PT; M	N/A	N/A	[48]
Colorectal cancer	SR	N/A	PT	81.5% (22/27)	N/A	[24]
Colorectal cancer	CT-guided NB	N/A	M	71% (10/14)	N/A	[49]
Colorectal cancer	SR; EB	N/A	PT; M	N/A	≥3 months	[18]
Colorectal cancer	EB; US-guided NB; CT-guided NB	N/A	M	N/A	N/A	[43]
Colorectal cancer	CT-guided NB	N/A	M ^h^	63% (40/63)	N/A	[50]
Colon adenoma	EB	Tubular adenoma; Tubulovillous adenoma; Sessilie serrated adenoma/polyp	PT	N/A	≥3 months	[18]
Rectal cancer	EB	Adenocarcinoma; Mucinous adenocarcinoma; Signet ring cell carcinoma	PT	85.7% (96/112)	N/A	[51]
Rectal cancer	SR; EB	N/A	PT; M ^i^	77% (65/84)	N/A	[52]

Abbreviations: Sample: SR, surgical resection; EB, endoscopic biopsy; FNA, Fine needle aspiration; US-guided NB, ultrasound-guided needle biopsy; CT-guided NB, computed tomography-guided needle biopsy. Cell Type: EADC, esophageal adenocarcinoma; ESCC, esophageal squamous cell carcinoma; PDAC, pancreatic ductal adenocarcinoma; HCC, hepatocellular carcinoma; CC, cholangiocarcinoma; CHC, combined hepatocellular-cholangiocarcinoma; LEL-CC, lymphoepithelioma-like cholangiocarcinoma; IPMN, intraductal papillary mucinous neoplasm; ACC, acinar cell carcinoma; MSI, microsatellite instability; EBV, Epstein–Barr virus; CIN, chromosome instability; GS, genomically stable. Location: PT, primary tumor; M, metastasis. Footnotes: ^a^: 8/10 for surgical resection; ^b^: 2/2 for fine needle aspiration; ^c^: 61/78 for surgical resection; ^d^: 43/60 for fine needle biopsy; ^e^: 9/20 for rapid autopsy; ^f^: including stomach corpusantrum carcinoma, stomach corpusantrum carcinoma, and adenocarcinoma of the esophagogastric junction; ^g^: including liver metastasis, lung metastasis, omentum metastasis, and diaphragm metastasis; ^h^: including liver metastasis, peritoneum metastasis, omentum metastasis, lung metastasis, and lymph node metastasis; ^i^: including splenic metastasis and peritoneal metastasis.

**Table 2:** Organoid culture-condition of patient-derived organoids in gastrointestinal cancers.

Cancer Type	Embed	Base	Elements	Ref.
Esophageal cancer	BME-2	Advanced DMEM/F12	Penicillin/streptomycin, Primocin, HEPES, GlutaMAX, B27, N-acetylcysteine, Nicotinamide, Noggin, EGF, A83-01, FGF10, Wnt-3A, R-Spondin1, SB202190	[37]
Esophageal cancer	Matrigel	Advanced DMEM/F12	Penicillin/streptomycin, Neomycin, Antibiotic-antimycotic, Primocin, HEPES, GlutaMAX, B27, N-acetylcysteine, Noggin, EGF, Gastrin, A83-01, CHIR 99021, Wnt-3A, Rspondin1, SB202190	[38]
Esophageal cancer	Matrigel	Advanced DMEM/F12	Penicillin/streptomycin, HEPES, GlutaMAX, B27, N-2, N-acetylcysteine, Nicotinamide, Noggin, EGF, Gastrin, A83-01, Wnt-3A, R-Spondin1, SB202190, Y-27632	[39]
Gastric cancer	Matrigel	Advanced DMEM/F12	Penicillin/streptomycin, HEPES, GlutaMAX, B27, N-acetylcysteine, Nicotinamide, Noggin, EGF, Gastrin, A-83-01, Y-27632, FGF10, Wnt-3A, R-Spondin1	[40]
Gastric cancer	Matrigel	Advanced DMEM/F12	Penicillin/streptomycin, Primocin, HEPES, GlutaMAX, B27, N-acetylcysteine, Noggin, EGF, Gastrin, A-83-01, Y-27632, FGF10, Wnt-3A, R-Spondin1, Nutlin3a ^b^	[41]
Gastric cancer	Matrigel	Advanced DMEM/F12	Penicillin/streptomycin, Primocin, HEPES, GlutaMAX, B27, N-acetylcysteine, EGF, Gastrin, A83-01, FGF10, Y-27632, Wnt-3A, R-Spondin1 ^c^	[42]
Gastric cancer	Matrigel	Advanced DMEM/F12	Penicillin-streptomycin, L-Glutamine, B27, N-2, Nicotinamide, Noggin, Gastrin, A83-01, R-Spondin1, Y-27632, PGE2, Wnt-3A, R-Spondin1, SB202190, BSA, EGF, FGF10, FGF-basic	[43]
Liver cancer	BME-2	Advanced DMEM/F12	Penicillin/streptomycin, HEPES, GlutaMAX, B27, N-2, N-acetylcysteine, Nicotinamide, EGF, Gastrin, A83-01, FGF10, Y-27632, FGF10, HGF, Forskolin, Dexamethasone	[44]
Liver cancer	Matrigel BME-2	Advanced DMEM/F12	Penicillin/streptomycin, Primocin, HEPES, GlutaMAX, B27, N-2, N-acetylcysteine, Nicotinamide, Noggin, EGF, Gastrin, A83-01, Y-27632, FGF10, HGF, Forskolin, Wnt-3A, R-Spondin1	[27,28]
Pancreatic cancer	Matrigel	Advanced DMEM/F12	Penicillin/streptomycin, Primocin, HEPES, GlutaMAX, B27, N-acetylcysteine, Nicotinamide, Noggin, EGF, Gastrin, A83-01, Y-27632, FGF10, Wnt-3A, R-Spondin1	[45]
Pancreatic cancer	Matrigel	Advanced DMEM/F12	Penicillin/streptomycin, Primocin, HEPES, GlutaMAX, B27, N-acetylcysteine, Nicotinamide, Noggin, EGF, Gastrin, A83-01, Y-27632, FGF10, Wnt-3A, R-Spondin1, PGE2	[46]
Pancreatic cancer	BME-2	Advanced DMEM/F12	Penicillin/streptomycin, Primocin, HEPES, GlutaMAX, B27, N-acetylcysteine, Nicotinamide, Noggin, EGF, Gastrin, A83-01, Y-27632, FGF10, Wnt-3A, R-spondin1 ^d^	[47]
Pancreatic cancer	Matrigel	Advanced DMEM/F12	Penicillin/streptomycin, HEPES, GlutaMAX, B27, N-acetylcysteine, Noggin, Gastrin, A83-01, Y27632, Wnt-3A, R-spondin1, SB202190	[48]
Colorectal cancer	BME-2 Matrigel	Advanced DMEM/F12	Penicillin/streptomycin, Primocin, HEPES, GlutaMAX, B27, N-2, N-acetylcysteine, Nicotinamide, Noggin, EGF, Gastrin, A83-01, Y-27632, PGE2, R-Spondin1, SB202190	[24,49]
Colorectal cancer and colon adenoma	Matrigel	Advanced DMEM/F12	Penicillin/streptomycin, HEPES, GlutaMAX, B27, N-acetylcysteine, Nicotinamide, Noggin, EGF, Gastrin, A83-01, Y-27632, Wnt-3A, R-Spondin1, SB202190 ^a^	[18]
Colorectal cancer	Matrigel	Advanced DMEM/F12	Penicillin/streptomycin, L-Glutamine, B27, N-2, Nicotinamide, Noggin, Gastrin, A83-01, R-Spondin1, Y-27632, PGE2, Wnt-3A, R-Spondin1, SB202190, BSA, EGF, FGF10, FGF-basic	[43]
Colorectal cancer	Matrigel	Advanced DMEM/F12	Penicillin/streptomycin, HEPES, GlutaMAX, B27, N-2, N-acetylcysteine, EGF, A-83-01, Y-27632, SB202190	[50]
Rectal cancer	Matrigel	Advanced DMEM/F12	Gentamicin/amphoteritin B, Normocin, HEPES, Glutamax B27, N-2, N-acetylcysteine, Nicotinamide, Noggin, EGF, Gastrin, A83-01, Y-27632, PGE2, R-Spondin1, SB202190	[51]
Rectal cancer	Matrigel	Advanced DMEM/F12	Antibiotic-antimycotic, HEPES, GlutaMAX, B27, N-2, N-acetylcysteine, Nicotinamide, EGF, Gastrin, A83-01, SB202190	[52]

Abbreviations: DMEM, Dulbecco’s Modified Eagle’s Medium; HEPES, N-2-hydroxyethylpiperazine-N-ethane-sulphonicacid; BME, basement membrane extract type 2; EGF, epidermal growth factor; PGE2, prostaglandin E2; BSA, bovine serum albumin; FGF, fibroblast growth factor; HGF, hepatocyte growth factor. Footnotes: ^a^: Wnt-3A, R-Spondin1, and SB202190 are optional, and organoids are separately incubated at 20% O_2_ and at 1% O_2_. ^b^: Nutlin3a is added for the enrichment of the tumor organoids carrying TP53 mutations. ^c^: For the enrichment of the GC organoids, 1-week treatment with Nutlin-3, culturing in the absence of A83-01 and presence of TGF-β, and culturing in the absence of EGF and F-GF10 were used. ^d^: Have two kinds of tumor medium. One is the absence of PGE2 and EGF, the other is the absence of PGE2, A83-01, and Wnt-3A.

**Table 3:** Advantages and limitations of three mainstream preclinical cancer models for drug screening [14,16,53,54,55,56,57,58,59].

Features	Cell Lines	Patient-Derived Xenografts	Patient-Derived Organoids
Cost	Low	Highest	Moderate
Ease of maintenance	Easy to culture	Must be maintained in immunocompromised mice	Variable success rate, easy to culture after establishment
Time demand	Low	Highest, serial passages in mice required	Moderate, faster than PDXs
Long-term stability	Moderate	High	High
In vivo tumor phenotype	Limited predictive power	Highest predictive power with modelling of stromal interactions	High predictive power
Tumor genomic spectrum	Low	High degree of tumor heterogeneity	Some degree of tumor heterogeneity
High throughput	Readily adopted for high-throughput assays	Not suitable for high-throughput assays	Can be adopted for high-throughput assays
Genetic manipulation	Easy	Difficult	Moderate

Abbreviations: PDXs, Patient-Derived Xenografts.
